# The Spinal Cord Expression of Neuronal and Inducible Nitric Oxide Synthases and Their Contribution in the Maintenance of Neuropathic Pain in Mice

**DOI:** 10.1371/journal.pone.0014321

**Published:** 2010-12-13

**Authors:** Arnau Hervera, Roger Negrete, Sergi Leánez, Jesús M. Martín-Campos, Olga Pol

**Affiliations:** 1 Grup de Neurofarmacologia Molecular, Institut de Recerca de l'Hospital de la Sta Creu i Sant Pau & Institut de Neurociències, Universitat Autònoma de Barcelona, Barcelona, Spain; 2 Grup de Bioquímica, Institut de Recerca de l'Hospital de la Sta Creu i Sant Pau, Barcelona, Spain; University of North Dakota, United States of America

## Abstract

**Background:**

Nitric oxide generated by neuronal (NOS1), inducible (NOS2) or endothelial (NOS3) nitric oxide synthases contributes to pain processing, but the exact role of NOS1 and NOS2 in the maintenance of chronic peripheral neuropathic pain as well as the possible compensatory changes in their expression in the spinal cord of wild type (WT) and NOS knockout (KO) mice at 21 days after total sciatic nerve ligation remains unknown.

**Methodology/Principal Findings:**

The mechanical and thermal allodynia as well as thermal hyperalgesia induced by sciatic nerve injury was evaluated in WT, NOS1-KO and NOS2-KO mice from 1 to 21 days after surgery. The mRNA and protein levels of NOS1, NOS2 and NOS3 in the spinal cord of WT and KO mice, at 21 days after surgery, were also assessed. Sciatic nerve injury led to a neuropathic syndrome in WT mice, in contrast to the abolished mechanical allodynia and thermal hyperalgesia as well as the decreased or suppressed thermal allodynia observed in NOS1-KO and NOS2-KO animals, respectively. Sciatic nerve injury also increases the spinal cord expression of NOS1 and NOS2 isoforms, but not of NOS3, in WT and NOS1-KO mice respectively. Moreover, the presence of NOS2 is required to increase the spinal cord expression of NOS1 whereas an increased NOS1 expression might avoid the up-regulation of NOS2 in the spinal cord of nerve injured WT mice.

**Conclusions/Significance:**

These data suggest that the increased spinal cord expression of NOS1, regulated by NOS2, might be responsible for the maintenance of chronic peripheral neuropathic pain in mice and propose these enzymes as interesting therapeutic targets for their treatment.

## Introduction

Neuropathic pain is a clinical manifestation characterized by the presence of exaggerated response to painful stimuli (hyperalgesia), pain response to normally innocuous stimuli (allodynia), and spontaneous pain. It is well accepted that nitric oxide synthesized by three nitric oxide synthases (neuronal, NOS1; inducible, NOS2 and endothelial, NOS3) regulates several cellular processes, such as pathological pain [1]–[2]. Therefore, several works using pharmacological and genetic approaches have demonstrated that selective NOS1 and NOS2 inhibitors might reverse the mechanical hypersensitivity to pain induced by spinal and peripheral neuropathy [3]–[5]. However and besides that these studies suggest a potential role of nitric oxide in the modulation of nerve injury-induced mechanical hypersensitivity, the exact contribution of NOS1 and NOS2 in the modulation of the thermal hyperalgesia and thermal allodynia induced by sciatic nerve injury remains unknown. Thus, the first aim of our study is to compare the pain-related behavior induced by the chronic constriction of the sciatic nerve in NOS1-KO, NOS2-KO and WT mice from days 1 to 21 after nerve injury.

Several works have demonstrated that nerve injury after sciatic nerve ligation evoked an increased NOS1 and NOS2, but not of NOS3, protein expression in the ipsilateral site of the dorsal root ganglia and the sciatic nerve [4], [6]–[11]. In contrast, the possible changes induced by peripheral neuropathic pain in the spinal cord expression of these genes remain controversial. That is, from no changes to an increased NOS protein expression in the spinal cord of sciatic nerve-injured animals at days 7^th^ to 26^th^ after surgery have been reported [4], [7]–[8], [11]. Therefore, our subsequent aim is to evaluate the mRNA and protein expression of NOS1 and NOS2 isoenzymes in the spinal cord of WT mice at 21 days after sciatic nerve ligation and correlate it with their corresponding behavior responses.

It is well known that the expression of other NOS isoforms is up-regulated in a compensatory manner in the spinal cord of NOS1 and NOS2 knockout mice under basal and inflammatory pain conditions [12]–[14]. However, the possible changes in the expression of NOS1, NOS2 and NOS3 isoforms that might occurs in the spinal cord of NOS1 and NOS2 knockout mice after total sciatic nerve ligation remains unknown. Thus, the mRNA and protein levels of these three isoenzymes (NOS1, NOS2 and NOS3) in the spinal cord of NOS2-KO and NOS1-KO mice at day 21 after sciatic nerve injury were also evaluated.

## Results

### Effect of NOS1 and NOS2 deletion on the development of neuropathic pain in mice

#### Mechanical allodynia

**Figure 1:**
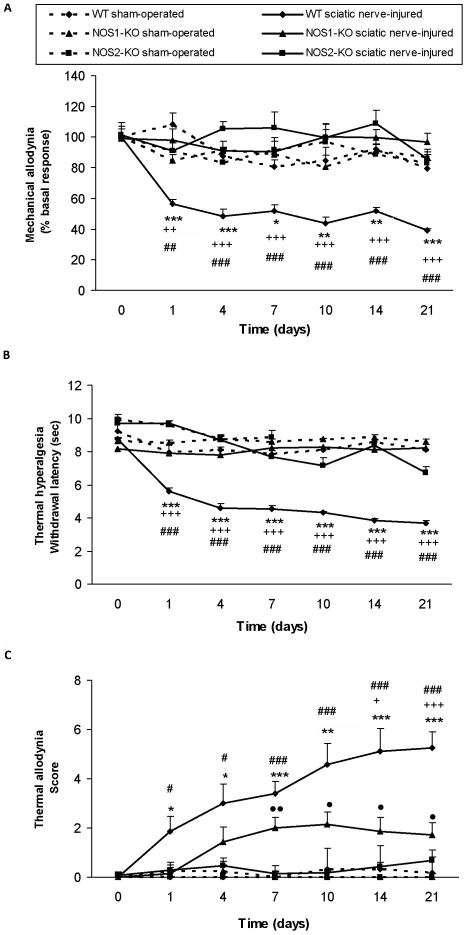
Effect of NOS1 and NOS2 deletion on sciatic nerve injury induced mechanical allodynia, thermal hyperalgesia and thermal allodynia in mice. Development of mechanical allodynia (**A**), thermal hyperalgesia (**B**) and thermal allodynia (**C**) in the ipsilateral paw of WT, NOS1-KO and NOS2-KO mice at 0, 1, 4, 7, 10, 14 and 21 days after sciatic nerve ligation. For each test and time tested, * indicates significant differences when compared sciatic nerve-injured WT mice vs. sham-operated WT mice (* p<0.05, ** p<0.01, *** p<0.001, one-way ANOVA followed by Scheffe test), + when compared sciatic nerve-injured NOS1-KO mice vs. sciatic nerve-injured WT mice (+ p<0.05, ++ p<0.01, +++ p<0.001, one-way ANOVA followed by Scheffe test) and # when compared sciatic nerve-injured NOS2-KO mice vs. sciatic nerve-injured WT mice (# p<0.05, ## p<0.01, ### p<0.001, one-way ANOVA followed by Scheffe test). In the cold plate test (C), • indicates significant differences when compared sciatic nerve-injured NOS1-KO mice vs. sham-operated NOS1-KO mice (• p<0.05, •• p<0.01, one-way ANOVA followed by Scheffe test). Results are shown as mean values ± SEM; n = 10–12 animals per experimental group.

Sciatic nerve ligature led to a significant decrease of the threshold for evoking paw withdrawal to a mechanical stimulus only in WT mice and this response was abolished in mice lacking NOS1 or NOS2 (Fig. 1A). Baseline values were similar in all genotypes. Sham operation did not produce any modification of the nociceptive responses in WT, NOS1-KO or in NOS2-KO mice for the whole duration of the experiment.

For NOS1-KO mice, the two-way ANOVA revealed a significant effect of the genotype (p<0.002) at days 4, 10, 14 and 21 after surgery, a significant effect of surgery (p<0.008) on days 1, 4, 7, 14 and 21 and a significant interaction between genotype and surgery from day 1 to day 21 (p<0.030) after surgery in the ipsilateral paw of NOS1-KO mice as compared to WT group.

In WT mice, nerve injury led to a significant decrease of the threshold for evoking hind paw withdrawal to a mechanical stimulus on the injured side. This mechanical allodynia appeared on the first measurement after sciatic nerve ligation (day 1) and persisted for the whole duration of the experiment. Indeed, a significant effect of sciatic nerve ligation was revealed on day 1 (p<0.001), day 4 (p<0.001), day 7 (p<0.039), day 10 (p<0.017), day 14 (p<0.007) and day 21 (p<0.001) after surgery (one-way ANOVA vs. WT sham-operated animals). In contrast, a significant decrease of mechanical allodynia was observed in NOS1-KO mice on day 1 (p<0.008), day 4 (p<0.001), day 7 (p<0.001), day 10 (p<0.001), day 14 (p<0.001) and day 21 (p<0.001) when compared to the WT group (one-way ANOVA).

The possible development of mechanical allodynia in NOS2-KO mice after sciatic nerve injury has been also evaluated. The two-way ANOVA revealed a significant effect of the genotype (p<0.001) at days 4, 7, 10, 14 and 21 after surgery, a significant effect of the surgery (p<0.023) on days 1, 10 and 21 and a significant interaction between genotype and surgery from day 1 to day 21 (p<0.008) after surgery in the ipsilateral paw of NOS2-KO mice as compared to WT group. Indeed, a significant decrease of mechanical allodynia was also observed in NOS2-KO mice on day 1 (p<0.008), day 4 (p<0.001), day 7 (p<0.001), day 10 (p<0.001), day 14 (p<0.001) and day 21 (p<0.001) when compared to WT group (one-way ANOVA).

Withdrawal latencies of the contralateral paw were not modified in any experimental group (data not shown).

#### Thermal hyperalgesia

Sciatic nerve ligature decreased paw withdrawal latency to thermal stimulus only in WT mice and this response was abolished in mice lacking NOS1 or NOS2 genes (Fig. 1B). Baseline values were similar in all genotypes. Sham operation did not produce any modification of nociceptive responses in WT, NOS1-KO or in NOS2-KO mice for the whole duration of the experiment.

Regarding NOS1-KO mice, the two-way ANOVA revealed a significant effect of the genotype (p<0.001), surgery (p<0.001) and their interaction (p<0.005) from day 1 to day 21 after surgery in the ipsilateral paw of NOS1-KO mice as compared to the WT group. Thus, a marked and long-lasting decrease of the paw withdrawal latencies was observed in the ipsilateral paw of WT mice exposed to sciatic nerve injury from day 1 to day 21 (p<0.001) after surgery (one-way ANOVA vs. WT sham-operated animals). In contrast, a significant decrease of thermal hyperalgesia was observed in sciatic nerve-injured NOS1-KO mice from day 1 to day 21 (p<0.001) when compared to the WT group (one-way ANOVA).

The possible development of thermal hyperalgesia in NOS2-KO mice after sciatic nerve injury has been also evaluated. The two-way ANOVA also revealed a significant effect of the genotype (p<0.001), surgery (p<0.001) and their interaction (p<0.007) from day 1 to day 21 after surgery in the ipsilateral paw of NOS2-KO mice as compared to WT group. Indeed, a significant decrease of thermal hyperalgesia was observed in sciatic nerve-injured NOS2-KO mice from day 1 to day 21 (p<0.001) when compared to the WT group (one-way ANOVA).

Withdrawal latencies of the contralateral paw were not modified in any of the experimental groups (data not shown).

#### Thermal allodynia

Sciatic nerve ligature enhanced the score values during the cold thermal stimulation in WT mice and this response was significantly attenuated or abolished in NOS1-KO and NOS2-KO mice (Fig. 1C), respectively. Baseline score values were similar in all genotypes. Sham operation did not produce any modification of thermal nociceptive responses neither in WT nor in KO mice for the whole duration of the experiment.

For NOS1-KO mice, the two-way ANOVA only revealed a significant effect of the genotype (p<0.029) at days 14 and 21 after surgery, a significant effect of the surgery (p<0.006) on days 4, 7, 10, 14 and 21 and a significant interaction between theme at days 1 and 21 (p<0.036) after surgery in the ipsilateral paw of NOS1-KO mice as compared to the WT group. Thus, WT mice exposed to sciatic nerve injury significantly increased the score values after surgery, revealing the development of thermal allodynia on day 1 (p<0.046), day 4 (p<0.028), day 7 (p<0.001), day 10 (p<0.004), day 14 (p<0.001) and day 21 (p<0.001) after surgery (one-way ANOVA vs. WT sham-operated animals). In NOS1-KO mice exposed to sciatic nerve injury only showed significant thermal allodynia on day 7 (p<0.008), day 10 (p<0.017), day 14 (p<0.050) and day 21 (p<0.011) after surgery (one-way ANOVA vs. sham operated animals), although a significant decrease of this thermal allodynia was observed when compared sciatic nerve-injured NOS1-KO vs. WT mice on days 14 (p<0.017) and 21 (p<0.001) after surgery (one-way ANOVA).

The possible development of thermal allodynia in NOS2-KO mice after sciatic nerve injury has been also evaluated. The two-way ANOVA revealed a significant effect of the genotype (p<0.011) from days 1 to 21 after surgery, a significant effect of surgery (p<0.001) on days 7, 10, 14 and 21 and a significant interaction between genotype and surgery from day 7 to day 21 (p<0.001) after surgery in the ipsilateral paw of NOS2-KO mice as compared to the WT group. Thus, thermal allodynia was not developed in NOS2-KO mice. Indeed, a significant decrease of thermal allodynia was observed when compared sciatic nerve-injured NOS2-KO mice on day 1 (p<0.049), day 4 (p<0.033), day 7 (p<0.001), day 10 (p<0.001), day 14 (p<0.001) and day 21 (p<0.001) with WT mice (one-way ANOVA).

### Expression of NOS1 in the spinal cord of WT and NOS2-KO mice

**Figure 2:**
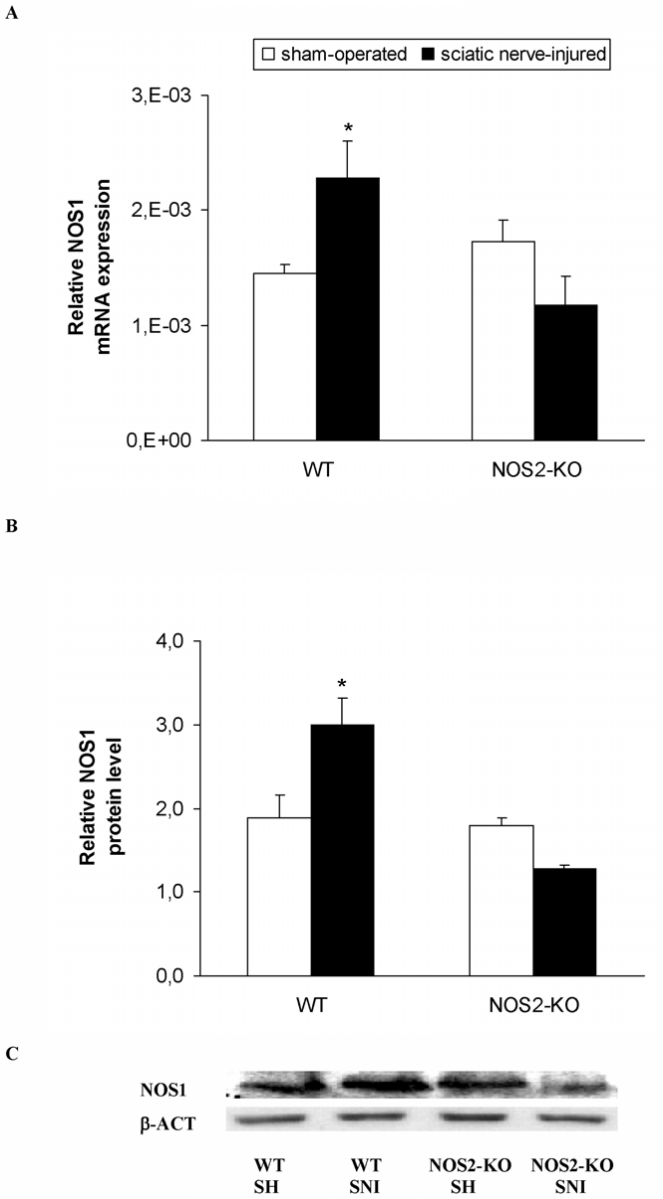
Spinal cord expression of NOS1 in WT and NOS2-KO mice. The relative NOS1 mRNA (**A**) and protein (**B**) expression in the ipsilateral site of the lumbar section of the spinal cord from sham-operated (SH) and sciatic nerve-injured (SNI) WT and NOS2-KO mice at 21 days after sciatic nerve ligation are represented. In both figures, * indicates significant differences when compared sciatic nerve-injured WT mice vs. sham-operated WT animals (* p<0.05, one-way ANOVA followed by Student-Newman-Keuls test). A representative example of Western blots for NOS1 protein (155 kDa) in which β-actin (43 kDa) was used as a loading control is shown in **C**. Data are expressed as mean values ± SEM; n = 4–5 samples per group.

The relative mRNA levels of NOS1 gene in the spinal cord from sham-operated and sciatic nerve-injured WT and NOS2-KO mice are shown in Fig. 2A. Our results showed that although the two way ANOVA did not show a significant effect of the genotype or surgery, a significant interaction between theme was demonstrated (p<0.005). Thus, sciatic nerve ligation significantly increased the NOS1 gene expression in WT but not in NOS2-KO mice, when comparing sham-operated vs. sciatic nerve-injured animals (one way ANOVA, p<0.016). Our results did not show any significant differences between genotypes as comparing the expression of NOS1 mRNA among theme in sham-operated mice.

The protein expression of NOS1 in the spinal cord from sham-operated and sciatic nerve-injured WT and NOS2-KO mice are shown in Fig. 2B. The two way ANOVA revealed a significant effect of the genotype (p<0.001) and a significant interaction between genotype and surgery (p<0.003). Thus and according to that occurs with the mRNA levels, while sciatic nerve ligation significantly increases the NOS1 protein levels in WT mice it did not change their expression in NOS2-KO animals, when comparing sham-operated vs. sciatic nerve-injured animals (one way ANOVA, p<0.001). Our results did not show any significant differences between genotypes when comparing the expression of NOS1 protein among theme in sham-operated mice.

The mRNA and protein levels of NOS1 gene in the spinal cord from sham-operated and sciatic nerve-injured NOS1-KO were undetectable (data not shown).

### Expression of NOS2 in the spinal cord of WT and NOS1-KO mice

**Figure 3:**
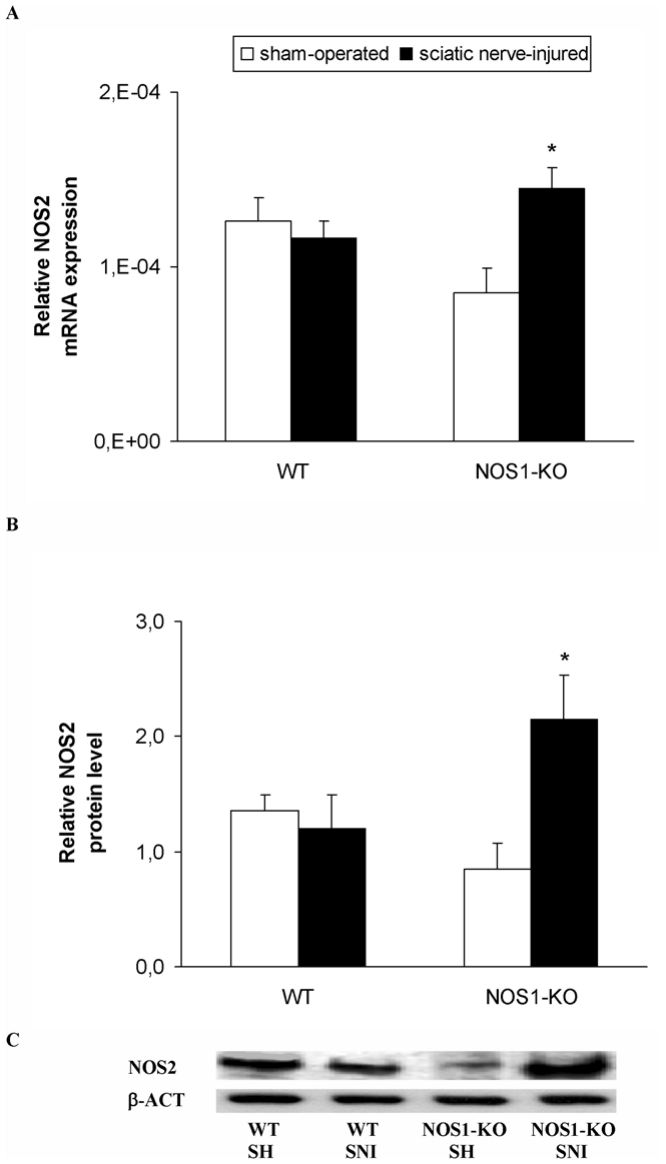
Spinal cord expression of NOS2 in WT and NOS1-KO mice. The relative NOS2 mRNA (**A**) and protein (**B**) expression in the ipsilateral site of the lumbar section of the spinal cord from sham-operated (SH) and sciatic nerve-injured (SNI) WT and NOS1-KO mice at 21 days after sciatic nerve ligation are represented. In both figures, * indicates significant differences when compared sciatic nerve-injured NOS1-KO mice vs. sham-operated NOS1-KO animals (* p<0.05, one-way ANOVA followed by Student-Newman-Keuls test). A representative example of Western blots for NOS2 protein (130 kDa) in which β-actin (43 kDa) was used as a loading control is shown in **C**. Data are expressed as mean values ± SEM; n = 4–5 samples per group.

The relative mRNA levels of NOS2 gene in the spinal cord from sham-operated and sciatic nerve-injured WT and NOS1-KO mice are shown in Fig. 3A. For NOS2 although the two way ANOVA did not show any effect of the genotype or surgery, a significant interaction between theme was demonstrated (p<0.011). Indeed, sciatic nerve ligation did not alter the NOS2 expression in the spinal cord of WT mice, but significantly increased their expression in NOS1-KO mice, when comparing sham-operated vs. sciatic nerve-injured animals (one way ANOVA, p<0.021). Non significant differences were found between genotypes as compared the expression of NOS2 mRNA among theme in sham-operated mice.

The protein expression of NOS2 in the spinal cord from sham-operated and sciatic nerve-injured WT and NOS1-KO mice are shown in Fig. 3B. The two way ANOVA did not show any effect of the genotype or surgery but a significant interaction between theme was demonstrated (p<0.019). Indeed, sciatic nerve injury did not alter the NOS2 protein levels in the spinal cord of WT mice but significantly increased their expression in NOS1-KO mice when comparing sham-operated vs. sciatic nerve-injured animals (one way ANOVA, p<0.028). Non significant differences were found between genotypes when compared the expression of NOS2 protein among theme in sham-operated mice.

The mRNA and protein levels of NOS2 gene in the spinal cord from sham-operated and sciatic nerve-injured NOS2-KO were undetectable (data not shown).

### Expression of NOS3 in the spinal cord of WT, NOS2-KO and NOS1-KO mice

**Figure 4:**
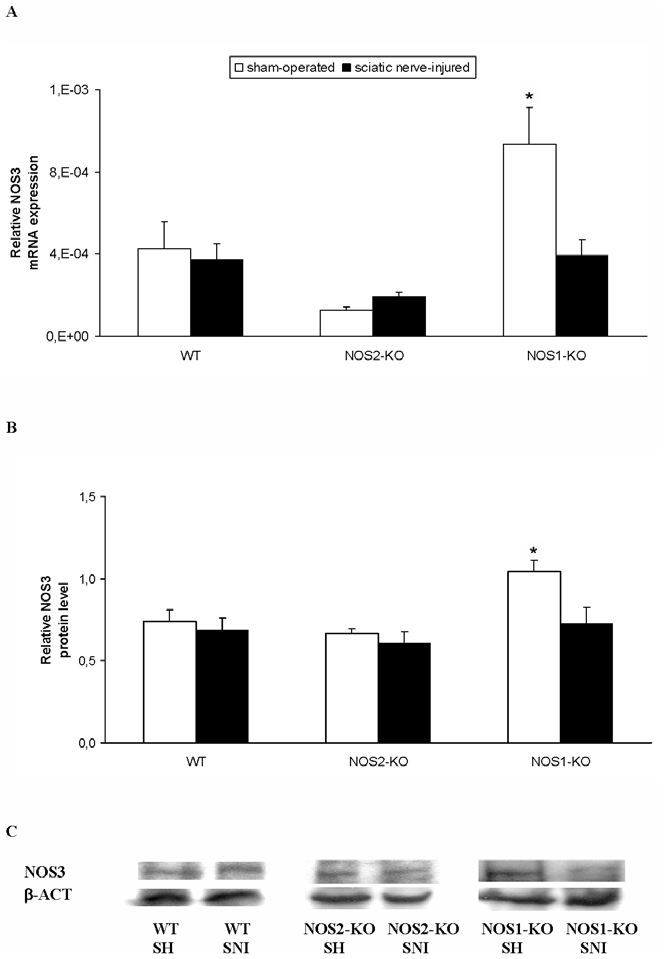
Spinal cord expression of NOS3 in WT, NOS2-KO and NOS1-KO mice. The relative NOS3 mRNA (**A**) and protein (**B**) expression in the ipsilateral site of the lumbar section of the spinal cord from sham-operated (SH) and sciatic nerve-injured (SNI) WT, NOS2-KO and NOS1-KO mice at 21 days after sciatic nerve ligation are represented. In both figures, * indicates significant differences when compared sham-operated NOS1-KO animals vs. the other groups. (* p<0.05, one-way ANOVA followed by Student-Newman-Keuls test). A representative example of Western blot for NOS3 protein (140 kDa) in which β-actin (43 kDa) was used as a loading control is shown in **C**. Data are expressed as mean values ± SEM; n = 4–5 samples per group.

The relative mRNA levels of NOS3 gene in the spinal cord from sham-operated and sciatic nerve-injured WT, NOS2-KO and NOS1-KO mice are shown in Fig. 4A. The two way ANOVA showed a significant effect of the genotype (p<0.001) and a significant interaction between genotype and surgery (p<0.016). Indeed, our results showed that while the spinal cord expression of NOS3 in sham-operated NOS1-KO mice was significantly higher than those presented in WT and NOS2-KO mice (one way ANOVA, p<0.005), non significant differences between genotypes were found at 21 days after nerve injury.

The protein levels of NOS3 in the spinal cord from sham-operated and sciatic nerve-injured WT, NOS2-KO and NOS1-KO mice are shown in Fig. 4B. The two way ANOVA showed a significant effect of the genotype (p<0.015) and surgery (p<0.034) and according to what occurs with the mRNA expression, while the NOS3 expression in sham-operated NOS1-KO mice was significantly higher to than those observed in sham-operated WT or NOS2-KO mice (one way ANOVA, p<0.006), these differences between genotypes disappear after sciatic nerve ligation.

## Discussion

The present study demonstrates for first time the participation of NOS1 and NOS2 in the development and maintenance of thermal hyperalgesia and thermal allodynia induced by the chronic constriction of sciatic nerve and suggests that the increased spinal cord expression of NOS1 is regulated by NOS2, and might be responsible for the maintenance of chronic peripheral neuropathic pain in mice. Our data also indicate that the enhanced spinal cord expression of NOS3 in NOS1-KO mice might compensates for the lack of NOS1 under basal conditions but not after sciatic nerve ligation.

Our results showed that the mechanical allodynia and thermal hyperalgesia induced by sciatic nerve injury was completely abolished in both NOS knockout mice, while thermal allodynia was significantly attenuated and completely abolished in NOS1-KO and NOS2-KO animals, respectively. In accordance to our results, the pharmacological inhibition of NOS1 or NOS2 isoenzymes as well as the genetic deletion of NOS1 both attenuate the mechanical hypersensitivity observed after the spinal or sciatic nerve injury in mice [4]–[5], [15]. Despite that, our study extended these findings and provides the first evidence that the targeted disruption of NOS1 or NOS2 genes also abolishes or diminishes the thermal hyperalgesia and thermal allodynia, the other major behavioral manifestations of neuropathic pain observed in WT mice from days 1 to 21 following sciatic nerve ligation. Before surgery, similar baseline withdrawal thresholds to mechanical and thermal stimuli were found in WT and both knockout mice. Responses of sham-operated mice also remained unchanged in NOS1 or NOS2 knockout mice as compared to WT. These results indicate that in the absence of nerve injury, the nitric oxide synthesized by NOS1 or NOS2 do not seems to tonically modulate the mechanical and thermal nociceptive sensitivity [13], [16]. Summing up, our data demonstrated that nitric oxide synthesized by both NOS1 and NOS2 isoenzymes is involved in the development and maintenance of sciatic nerve injury-induced neuropathic pain.

It is well known that nitric oxide generated by NOS1 or NOS2 also contributes to the processing of nociceptive signals induced by other types of chronic pain such as inflammatory [17]. In accordance, our and other previous studies have revealed the different roles played by nitric oxide synthesized by NOS1 or NOS2 in the maintenance of mechanical allodynia and thermal hyperalgesia induced by inflammatory pain [16], [18]. Surprisingly, the behavioral data of this study show that both enzymes (NOS1 and NOS2) have a similar participation in the expression of sciatic nerve injury-induced mechanical allodynia and thermal hyperalgesia. Thus, it is only in the maintenance of sciatic nerve injury-induced thermal allodynia where NOS2 seems to be more implicated than NOS1. These results support the hypothesis that the involvement of each NOS enzyme varies according to the type of nociceptive stimulus [3], [5], [19].

The molecular mechanisms implicated in the neuropathic pain states are not clear. However, one consequence of nerve injuries is the manifestation of adaptive changes in the expression of diverse receptors, channels and enzymes, such as NOS in the dorsal root ganglia and the spinal cord of nerve injured animals. At present, the possible effects of chronic sciatic nerve ligation on the induction of NOS enzymes in the spinal cord are not completely elucidated. Our data showed that peripheral neuropathic pain increases the transcription and expression of NOS1 in the spinal cord of WT mice, indicating that NOS1 might be the main responsible for the maintenance of peripheral neuropathic pain after the total sciatic nerve ligation. In accordance to our results, an increase in the spinal cord expression of NOS1 at 14 days after nerve injury have been also demonstrated although the expression of this isoenzyme on day 7^th^ after injury remains unaltered [5], [7]–[8]. These findings indicate that the NOS1 spinal cord changes induced by nerve injury could be related to the time point after surgery, suggesting that NOS1 seems to be more involved in the late (14 and 21 days) than in the early stages of sciatic nerve lesion.

The possible alterations in NOS2 and NOS3 expression induced by neuropathic pain have been also evaluated in this study. Our results did not show significant changes in the expression of NOS2 and NOS3, mRNA and protein, on day 21^th^ after sciatic nerve injury. In accordance to our findings, DeAlba et al. [4] also demonstrated that the spinal cord protein levels of NOS2 did not change at 26 days after total sciatic nerve ligation, although an increase in the protein levels of NOS2 has been also demonstrated in the spinal cord of WT mice at 7 or 14 days after nerve injury [8]. These data suggest that spinal NOS2 seems to be more involved in the early (7 and 14 days) than in the late (21 and 26 days) stages of nerve injury-induced neuropathic pain

In contrast to NOS1 and NOS2, the non significant changes in the spinal cord expression of NOS3 in the early (7 and 14 days) or late (21 days) phases of nerve injury suggest that this isoenzyme did not play an essential role in the development or maintenance of neuropathic pain [5], [11].

The possible compensatory changes in the expression of other NOS isoforms that might occurs in the spinal cord of NOS1 and NOS2 knockout mice at 21 days after sciatic nerve ligation were also evaluated. Our results demonstrate that in contrast to WT mice, the spinal cord expression of NOS1 in NOS2-KO animals was not altered by sciatic nerve injury. Therefore, we hypothesized that the unaltered changes in NOS1 expression observed in NOS2-KO mice and the genetic lack of NOS2 in these KO mice might explain the absence of mechanical and thermal allodynia as well as thermal hyperalgesia in their behavior responses after sciatic nerve ligation.

Our results also show, for first time, that the expression of NOS2 is up-regulated in the spinal cord of NOS1-KO mice during chronic sciatic nerve injury-induced neuropathic pain. This compensatory up-regulation of NOS2 in NOS1-KO mice suggests that the increased expression of this isoenzyme might be responsible for the thermal allodynia observed in NOS1-KO mice at 21 days after surgery. This hypothesis was supported by the absence of thermal allodynia observed in NOS2-KO mice at this time point after sciatic nerve ligation. In addition, our data also indicate that the presence of NOS2 is required for the increased expression of NOS1 in the spinal cord whereas an enhanced expression of NOS1 might avoid an up-regulation of NOS2 in the spinal cord of sciatic nerve-injured mice.

In the present study, we also demonstrated that no compensatory changes in the spinal cord expression of NOS3 take place in sciatic nerve-injured NOS1 and NOS2 knockout mice as well as in sham-operated NOS2-KO mice [12]. In addition, no compensatory changes in the spinal cord expression of NOS1 gene in sham-operated NOS2-KO mice have been also established. However, while the disruption of NOS1 gene did not alter the mRNA or protein levels of NOS2 in sham-operated mice a significant increase in the spinal cord expression of NOS3 was further demonstrated in sham-operated NOS1-KO mice [18]. These results indicated that the compensatory changes in the spinal cord expression of NOS3 in NOS1-KO under basal conditions does not fully compensate for NOS1 function in neuropathic pain, which is mainly produced by NOS2.

In summary, our results demonstrate the participation of nitric oxide synthesized by NOS1 or NOS2 in the development and maintenance of mechanical and thermal allodynia as well as the thermal hyperalgesia induced by the total constriction of sciatic nerve and propose these enzymes as interesting therapeutic targets for the treatment of chronic peripheral neuropathic pain. Our data also indicate that: i) the increased spinal cord expression of NOS1 plays a critical role in the maintenance of peripheral neuropathic pain, ii) a compensatory up-regulation of NOS2 isoform takes place in the spinal cord of sciatic nerve-injured NOS1-KO mice, and iii) the up-regulation of NOS1 in the spinal cord of sciatic nerve-injured WT mice at 21 days after surgery is modulated by NOS2.

## Materials and Methods

### Animals

Male NOS1-knockout mice (C57BL/6J background) and NOS2-knockout mice (C57BL/6J background) were purchased from Jackson Laboratories (Bar Harbor, ME, USA) and wild type (WT) mice with the same genetic background (C57BL/6J) were acquired from Harlan Laboratories (Barcelona, Spain). All mice weighing 21 to 25 g were housed under 12-h/12-h light/dark conditions in a room with controlled temperature (22°C) and humidity (66%). Animals had free access to food and water and were used after a minimum of 6 days acclimatization to the housing conditions. All experiments were conducted between 9:00 AM and 5:00 PM. Animal procedures were conducted in accordance with the guidelines of the European Communities, Directive 86/609/EEC regulating animal research and approved by the local ethical committee of our Institution (Comissió d'Etica en l'Experimentació Animal i Humana de la Universitat Autònoma de Barcelona, #00691).

### Induction of neuropathic pain

Neuropathic pain was induced by the chronic constriction of the sciatic nerve. Briefly, sciatic nerve ligation was performed under isoflurane anesthesia (3% induction, 2% maintenance). The biceps femoris and the gluteus superficialis were separated by blunt dissection, and the right sciatic nerve was exposed. The injury was produced by tying three ligatures around the sciatic nerve as described by Bennett [20]. The ligatures (4/0 silk) were tied loosely around the nerve with 1 mm spacing, until they elicited a brief twitch in the respective hindlimb, which was prevented from applying a too strong ligation, taking care to preserve epineural circulation. Sham-operated WT and KO mice have been used as controls.

The development of mechanical and thermal allodynia, and thermal hyperalgesia was evaluated by using the von Frey filaments, cold plate and plantar tests, respectively. All genotypes were tested in each paradigm on days 0, 1, 4, 7, 10, 14 and 21 after sciatic nerve ligation or sham induction.

### Nociceptive behavioural tests

**Mechanical allodynia** was quantified by measuring the hind paw withdrawal response to von Frey filament stimulation. In brief, animals were placed in a Plexiglas® box (20 cm high, 9 cm diameter) with a wire grid bottom through which the von Frey filaments (North Coast Medical, Inc., San Jose, CA, USA) bending force range from 0.008 to 3.5 g, were applied by using a modified version of the up–down paradigm, as previously reported by Chaplan et al. [21]. The filament of 0.4 g was used first and the 3.5 g filament was used as a cut-off. Then, the strength of the next filament was decreased or increased according to the response. The threshold of response was calculated from the sequence of filament strength used during the up–down procedure by using an Excel program (Microsoft Iberia SRL, Barcelona, Spain) that includes curve fitting of the data. Clear paw withdrawal, shaking or licking of the paw were considered nociceptive-like responses. Both ipsilateral and contralateral hind paws were tested. Animals were allowed to habituate for 1 h before testing in order to allow an appropriate behavioral immobility.

**Thermal hyperalgesia** was assessed as previously reported by Hargreaves et al. [22]. Paw withdrawal latency in response to radiant heat was measured using the plantar test apparatus (Ugo Basile, Italy). Briefly, mice were placed in Plexiglas boxes (20 cm high ×9 cm diameter) positioned on a glass surface. The heat source was positioned under the plantar surface of the hind paw and activated with a light beam intensity, chosen in preliminary studies to give baseline latencies from 8 to 10 s in control mice. A cut-off time of 12s was used to prevent tissue damage in absence of response. The mean paw withdrawal latencies from the ipsilateral and contralateral hind paws were determined from the average of 3 separate trials, taken at 5 min intervals to prevent thermal sensitization and behavioral disturbances. Animals were habituated to the environment for 1 h before the experiment to become quiet and to allow testing.

**Thermal allodynia** to cold stimulus was assessed by using the hot/cold-plate analgesia meter (Ugo Basile, Italy), previously described by Bennett and Xie [20]. The number of elevations of each hind paw was recorded in the mice exposed to the cold plate (4±0.5°C) for 5 minutes. For each animal, a score was calculated as the difference of number of elevations between ipsilateral and contralateral paw.

### Molecular experiments

#### Tissue isolation

Sham-operated and sciatic nerve-injured WT, NOS1-KO and NOS2-KO mice were sacrificed at 21 days after surgery by cervical dislocation. Tissues from the ipsilateral site of the lumbar section of the spinal cord were removed immediately after sacrifice, frozen in liquid nitrogen and stored at −80°C until assay. Because of the small size of the unilateral lumbar section of the spinal cord, tissues from two animals were pooled together to obtain enough RNA or protein levels for performing the real time-PCR or Western blot analysis.

#### Total RNA extraction and reverse transcription

Tissues were homogenized in ice-cold with a homogenizer (Ultra-Turf, T8; Ika Werke, Staufen, Germany) and the total RNA was extracted with TRIzol reagent (Invitrogen, Renfrewshire, England). The amount of the purified RNA (A_260_/A_280_ ratio was ≥1.9) was determined by spectrophotometry. In all experiments, 1 µg of total RNA was reverse transcribed into cDNA using SuperScript II RNAse H^-^ reverse transcriptase (Invitrogen, Renfrewshire, UK) in a final volume of 10 µl. Negative controls were performed in which all of the components were included except reverse transcriptase.

#### TaqMan probe real-time polymerase chain reaction (PCR)

The expression of NOS1, NOS2 and NOS3 was determined by relative real-time PCR using pre-developed mice TaqMan gene expression assays (Applied Biosystems, CA, USA) for these genes: Mm0435189_m1 for NOS1, Mm01309902_m1 for NOS2 and Mm00435217_m1 for NOS3. A probe against GAPDH (Mm 99999915_g1) was used as endogenous control. PCR reactions were set up in 96-well plates containing the corresponding cDNA, 2× universal master mix (Applied Biosystems, CA, USA), the forward and reverse primers and the TaqMan probe. The PCR reaction mixture also contained PCR buffer, MgCl_2_, dNTPs, and the thermal stable AmpliTaq Gold® DNA polymerase. The assay was conducted using the Applied Biosystems ABI PRISM 7000 Sequence Detection System. Water controls were included to ensure specificity. All samples were assayed in duplicate. Relative expression of the target genes was calculated by means of the comparative threshold cycle (CT) method [23].

#### Western blot analysis

The NOS1, NOS2 and NOS3 protein levels in the lumbar section of the spinal cord were analyzed by Western blot. Tissues were homogenized in buffer (50 mM Tris-Base, 150 nM NaCl, 1% NP-40, 2 mM EDTA, 1 mM phenylmethylsulfonyl fluoride, 0.5 Triton X-100, 0.1% SDS, 1 mM Na_3_VO_4_, 25 mM NaF, 0.5% protease inhibitor cocktail, 1% phosphatise inhibitor cocktail). All reactive were purchased at Sigma (St. Louis, MO, USA) with the exception of NP-40 from Calbiochem (Biosciences, La Jolla, CA, USA). The crude homogenate was solubilised 1 hour at 4°C, sonicated for 10 seconds and centrifugated at 4°C for 15 min at 700×g. The supernatants (100 µg of total protein) were mixed with 4× laemmli loading buffer and then loaded onto 4% stacking/5% separating SDS-polyacrylamide gels. The proteins were electrophoretically transferred onto PVDF membrane overnight, blocked with PBST +2% nonfat dry milk, and subsequently incubated overnight at 4°C with a polyclonal rabbit anti-NOS1 antibody (1∶150, BD Transduction Laboratories, San Diego, CA, USA), a polyclonal rabbit anti-NOS2 antibody (1∶200, Chemicon, Millipore), a polyclonal rabbit anti-NOS3 antibody (1∶100, BD Transduction Laboratories, San Diego, CA, USA), or a monoclonal rabbit anti-β-actin antibody (1∶10.000, Sigma, St. Louis, MO, USA). β-actin was used as a loading control. The proteins were detected by an horseradish peroxidase-conjugated anti-rabbit secondary antibody (GE Healthcare, Little Chalfont, Buckinghamshire, UK) and visualized by chemiluminescence reagents provided with the ECL kit (Amersham Pharmacia Biotech, Piscataway, NJ, USA) and exposure onto hyperfilm (GE, Healthcare). The intensity of blots was quantified by densitometry.

#### Experimental protocol

In a first set of experiments, we assessed the influence of NOS1 or NOS2 deletion in the development and expression of peripheral neuropathic pain. Then, WT, NOS1-KO and NOS2-KO mice were habituated for 1 h to the environment of the different experimental tests during 4 days. After the habituation period, baseline responses were established in the following sequence: von Frey filaments, plantar and cold plate tests. After baseline measurements neuropathic pain was induced as previously described. All animals were tested in each paradigm on days 1, 4, 7, 10, 14 and 21 after the chronic constriction of the sciatic nerve by using the same sequence as for baseline responses. We used sham-operated WT, NOS1-KO and NOS2-KO mice as controls.

In a second set of experiments, we examined the mRNA and protein levels of NOS1, NOS2 and NOS3 in the ipsilateral site of the lumbar section of the spinal cord from sciatic nerve-injured and sham-operated WT, NOS1-KO and NOS2-KO mice at 21 days after surgery by using real time PCR and Western blot, respectively.

#### Statistical analysis

Data are expressed as mean ± standard error of the mean (SEM). For each knockout mice and experimental day, data obtained in the von Frey filament stimulation model, plantar and cold plate tests from sciatic nerve-injured or sham-operated mice were compared by using a two-way ANOVA repeated measures (genotype and surgery as between factors of variation), followed by a one-way ANOVA or the corresponding Student's t test when required. Changes in the expression (mRNA and protein levels) of NOS1, NOS2 and NOS3 in the lumbar section of the spinal cord from sciatic nerve-injured or sham-operated WT, NOS1-KO and NOS2-KO mice at 21 days after surgery were analyzed by using a two-way ANOVA (genotype and surgery as between factors of variation), followed by a one-way ANOVA when required. A value of p<0.05 was considered significant.

## References

[1] Meller ST, Pechman PS, Gebhart GF, Maves TJ (1992). Nitric oxide mediates the thermal hyperalgesia produced in a model of neuropathic pain in the rat.. Neuroscience.

[2] Nathan C, Xie QW (1994). Regulation of biosynthesis of nitric oxide.. J Biol Chem.

[3] La Buda CJ, Koblish M, Tuthill P, Dolle RE, Little PJ (2006). Antinociceptive activity of the selective iNOS inhibitor AR-C102222 in rodent models of inflammatory, neuropathic and post-operative pain.. Eur J Pain.

[4] De Alba J, Clayton NM, Collins SD, Colthup P, Chessell I (2006). GW274150, a novel and highly selective inhibitor of the inducible isoform of nitric oxide synthase (iNOS), shows analgesic effects in rat models of inflammatory and neuropathic pain.. Pain.

[5] Guan Y, Yaster M, Raja SN, Tao Y-X (2007). Genetic knockout and pharmacologic inhibition of neuronal nitric oxide synthase attenuate nerve injury-induced mechanical hypersensitivity in mice.. Mol Pain.

[6] Levy D, Zochodne DW (1998). Local nitric oxide synthase activity in a model of neuropathic pain.. Eur J Neurosci.

[7] Valsecchi AE, Franchi S, Panerai AE, Sacerdote P, Trovato AE (2008). Genistein, a natural phytoestrogen from soy, relieves neuropathic pain following chronic constriction sciatic nerve injury in mice: anti-inflammatory and antioxidant activity.. J Neurochem.

[8] Martucci C, Trovato AE, Costa B, Borsani E, Franchi S (2008). The purinergic antagonist PPADS reduces pain related behaviours and interleukin-1 beta, interleukin-6, iNOS and nNOS overproduction in central and peripheral nervous system after peripheral neuropathy in mice.. Pain.

[9] Levy D, Höke A, Zochodne DW (1999). Local expression of inducible nitric oxide synthase in an animal model of neuropathic pain.. Neurosci Lett.

[10] Costa B, Trovato AE, Colleoni M, Giagnoni G, Zarini E (2005). Effect of the cannabinoid CB1 receptor antagonist, SR141716, on nociceptive response and nerve demyelination in rodents with chronic constriction injury of the sciatic nerve.. Pain.

[11] Levy D, Tal M, Höke A, Zochodne DW (2000). Transient action of the endothelial constitutive nitric oxide synthase (ecNOS) mediates the development of thermal hypersensitivity following peripheral nerve injury.. Eur J Neurosci.

[12] Tao F, Tao YX, Mao P, Zhao C, Li D (2003). Intact carrageenan-induced thermal hyperalgesia in mice lacking inducible nitric oxide synthase.. Neuroscience.

[13] Boettger MK, Üceyler N, Zelenka M, Schmitt A, Reif A (2007). Differences in inflammatory pain in nNOS-, iNOS- and eNOS-deficient mice.. Eur J Pain.

[14] Tao F, Tao YX, Zhao C, Doré S, Liaw WJ (2004). Differential roles of neuronal and endothelial nitric oxide synthases during carrageenan-induced inflammatory hyperalgesia.. Neuroscience.

[15] Tanabe M, Nagatani Y, Saitoh K, Takasu K, Ono H (2009). Pharmacological assessments of nitric oxide synthase isoforms and downstream diversity of NO signaling in the maintenance of thermal and mechanical hypersensitivity after peripheral nerve injury in mice.. Neuropharmacology.

[16] Leánez S, Hervera A, Pol O (2009). Peripheral antinociceptive effects of μ- and δ-opioid receptor agonists in NOS2 and NOS1 knockout mice during chronic inflammatory pain.. Eur J Pharmacol.

[17] Schmidtko A, Tegeder I, Geisslinger G (2009). No NO, no pain? The role of nitric oxide and cGMP in spinal pain processing.. Trends Neurosci.

[18] Chu YC, Guan Y, Skinner J, Raja SN, Johns RA (2005). Effect of genetic knockout or pharmacologic inhibition of neuronal nitric oxide synthase on complete Freund's adjuvant-induced persistent pain.. Pain.

[19] Pol O (2007). The involvement of the nitric oxide in the effects and expression of opioid receptors during peripheral inflammation.. Curr Med Chem.

[20] Bennett GJ, Xie YK (1988). A peripheral mononeuropathy in rat that produces disorders of pain sensation like those seen in man.. Pain.

[21] Chaplan SR, Bach FW, Pogrel JW, Chung JM, Yaksh TL (1994). Quantitative assessment of tactile allodynia in the rat paw.. J Neurosci Methods.

[22] Hargreaves K, Dubner R, Brown F, Flores C, Joris J (1988). A new and sensitive method for measuring thermal nociception in cutaneous hyperalgesia.. Pain.

[23] Livak KJ, Schmittgen TD (2001). Analysis of relative gene expression data using real-time quantitative PCR and the 2(-Delta Delta C(T)) method.. Methods.

